# NovelFam3000 – Uncharacterized human protein domains conserved across model organisms

**DOI:** 10.1186/1471-2164-7-48

**Published:** 2006-03-13

**Authors:** Danielle Kemmer, Raf M Podowski, David Arenillas, Jonathan Lim, Emily Hodges, Peggy Roth, Erik LL Sonnhammer, Christer Höög, Wyeth W Wasserman

**Affiliations:** 1 Center for Genomics and Bioinformatics, Karolinska Institutet, Stockholm, Sweden; 2 Centre for Molecular Medicine and Therapeutics, University of British Columbia, Vancouver, Canada; 3 Department of Developmental Biology, Stockholm University, Stockholm, Sweden; 4 Department of Medical Genetics, University of British Columbia, Vancouver, Canada

## Abstract

**Background:**

Despite significant efforts from the research community, an extensive portion of the proteins encoded by human genes lack an assigned cellular function. Most metazoan proteins are composed of structural and/or functional domains, of which many appear in multiple proteins. Once a domain is characterized in one protein, the presence of a similar sequence in an uncharacterized protein serves as a basis for inference of function. Thus knowledge of a domain's function, or the protein within which it arises, can facilitate the analysis of an entire set of proteins.

**Description:**

From the Pfam domain database, we extracted uncharacterized protein domains represented in proteins from humans, worms, and flies. A data centre was created to facilitate the analysis of the uncharacterized domain-containing proteins. The centre both provides researchers with links to dispersed internet resources containing gene-specific experimental data and enables them to post relevant experimental results or comments. For each human gene in the system, a characterization score is posted, allowing users to track the progress of characterization over time or to identify for study uncharacterized domains in well-characterized genes. As a test of the system, a subset of 39 domains was selected for analysis and the experimental results posted to the NovelFam3000 system. For 25 human protein members of these 39 domain families, detailed sub-cellular localizations were determined. Specific observations are presented based on the analysis of the integrated information provided through the online NovelFam3000 system.

**Conclusion:**

Consistent experimental results between multiple members of a domain family allow for inferences of the domain's functional role. We unite bioinformatics resources and experimental data in order to accelerate the functional characterization of scarcely annotated domain families.

## Background

The number of protein-encoding human genes identified has reached a plateau [1], leaving researchers with the challenging task of ascribing biochemical function(s) for each protein [2]. Broad genome sequencing and functional genomics studies, partially motivated by the goal to discover the functions of uncharacterized proteins, have provided a distributed set of data collections suitable to catalyze the inference of the functions of proteins. While gene predictions and high-throughput genomics data can be of variable quality, studies have demonstrated that consistent results for interactions between homologous genes in multiple organisms, so called Interolog Analysis, can be more reliable [3-5]. Therefore, human protein characterization efforts that focus on similar proteins across multiple organisms are expected to more effectively capitalize on the available genomics data.

The genome sequence annotation and functional genomics data of *Caenorhabditis elegans*, *Drosophila melanogaster*, and *Homo sapiens *(hereafter referred to as worm, fly, and human) provide the basis for the study of proteins conserved across metazoan species. In pursuing comparative genomics approaches for functional inference of protein function, the initial selection of related proteins separated by great evolutionary distances can be a challenge. A decision must often be drawn between the study of homologous and orthologous proteins. In addition to technical difficulties and controversies that can arise in ortholog identification, a conservative focus on the study of orthologs greatly limits the number of proteins available to study. For homolog studies, grouping full-length protein sequences by similarity is not always feasible. The modular evolution of proteins presents a systematic complication – unrelated pairs of proteins can be linked through additional proteins sharing a domain with each pair (e.g. a protein with domains A and B may be linked to a protein with domains C and D via an intermediary protein with domains B and C). This problem is ameliorated by placing the focus on modular protein domain families, in which proteins are linked by the presence of a common domain [6]. Resources are well established which describe protein domain families, including such examples as Pfam, InterPro, and Panther [7-9]. Those domains observed in proteins from multiple species are likely to be most reliable [10].

Characterization of protein function remains a fundamental challenge in functional genomics research. We have created the NovelFam3000 data centre to accelerate the study of uncharacterized domains conserved across worm, fly, and human. Building on domains identified in Pfam [7], we systematically link domain-containing proteins to functional genomics data in online databases. The NovelFam3000 system allows users to post both comments and experimental data. For a selected subset of the uncharacterized domain-containing families, we generate and post expression profiles and proteomic sub-cellular localization images. Specific examples are presented showing how a combination of experimental approaches and bioinformatics resources may elucidate functional characteristics of uncharacterized domains.

## Construction and content

### Selection of uncharacterized domain families

The characterization state of each protein domain is dynamic, dependent both on the available experimental literature and the perspective of the observing scientist. Using the Pfam database [7], we extracted approximately 3000 protein domain families for which we judged minimal biochemical annotation to be available (hence the name NovelFam3000). We limited our search to protein families present in genes from three metazoan genomes (worm, fly, and human), for which there were multiple human protein members. Applying these criteria, we extracted 2785 Pfam-B domain families and 127 families of Domains of Unknown Function (DUFs). The Pfam-B and DUF classes are distinguished by the level of human curation, as Pfam-B domains represent purely computational analysis and DUFs have been subjected to curator review. Of these domains, 892 (32%) of selected Pfam-B domains and 59 (46%) selected DUFs included at least one yeast protein member.

### NovelFam3000 system overview

**Figure 1:**
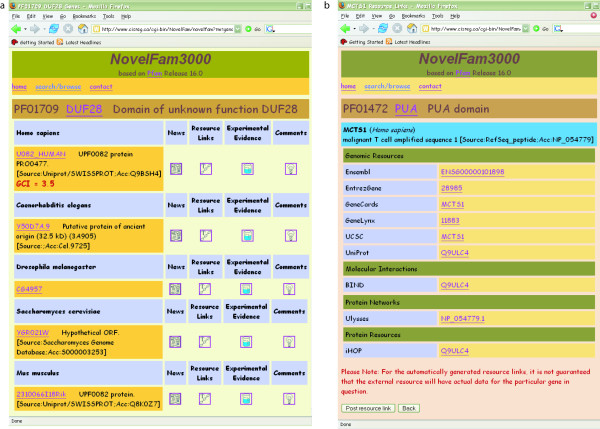
Screenshots of the NovelFam3000 interface. (a) The protein domain page displays protein members across model organisms for a given protein domain family. Detailed information about each gene is available via various hyperlinks including news, resource links, experimental evidence, and comments. Each link takes the user to a separate page with available link-specific information and the option to submit new data and comments. (b) The resource link displays hyperlinks to a set of bioinformatics databases containing information for a specific domain family member. For this human example, resources are divided into genomic resources, molecular interactions, and protein networks and resources. The user has the option to submit new resource links to the system.

For the selected domains, we constructed a database and an annotation system that unites links to bioinformatics resources with user-submitted experimental data to accelerate inference of domain function [11] (Figure 1) [see Additional file 1]. Users may query the database either with identifiers (for genes or domains) or sequence. A submitted protein sequence is analyzed with a Hidden Markov Model (HMM) search [12] to identify matches to DUFs included in NovelFam3000. Since there are currently no HMM models for Pfam-B domains, BLAST [13] searches are performed against the ProDom protein sequence database [14,15], and the detected ProDom identifiers are mapped to corresponding Pfam-B accessions. For clarity, Pfam-B domains are derived from a subset of domains present in the ProDom database. The HMM-detected DUF matches and the BLAST-detected Pfam-B matches are displayed as search results. Based on the input, the user is taken to a "domain page" from which all reported family members can be perused.

## Utility

### The NovelFam3000 annotation system

Users may view and post detailed information about each gene using four categories: i) resource links, linking to major bioinformatics resources, ii) news, highlighting the latest annotations submitted to the system, iii) comments, giving users the opportunity to view and post general comments regarding the domain-containing protein of interest, and iv) experimental evidence, displaying results submitted by individual researchers. At the bottom of each page displaying gene-specific information for one of the four categories, the user is prompted to submit new information. Submitted data are rendered accessible through the system within 24 hours, after brief editorial review to confirm relevance (i.e. to prevent posting of unrelated material).

For each gene, links to both diverse external resources and user-submitted experimental results and comments are provided via a "gene page". Organism-centric resource links for each gene include WormBase [16], Flybase [17], and SGD [18]. For human proteins, links are provided to genome browsers [19,20] and the meta-database GeneLynx [21]. For each protein, we provide links to the Biomolecular Interaction Network Database (BIND) [22], as well as to the Interolog Analysis system Ulysses [5,23] that displays protein-protein interactions observed for homologous proteins across fly, worm, human, and yeast.

Within the NovelFam3000 system, we report the Gene Characterization Index (GCI) for each human gene, providing users with a measure of the current knowledge of the gene's function. GCI scores assign a continuous score in the range of one (uncharacterized) to ten (fully characterized). The GCI system (Podowski *et al*., in preparation) is based on the results of a global survey of research biologists. Each participating scientist was given a sample of ten genes and returned their opinion as to the characterization status. The survey covered a total set of 100 genes with at least three fold redundancy. A machine learning procedure was used to create a scoring function to automatically predict the GCI score for all genes in the human genome. In this step, a Support Vector Machine was trained based on the survey results as training data, and the number of links to common databases (e.g. links to abstracts in PubMed or domains in Pfam).

Both the gene-specific news and user comment features allow researchers to highlight recent publications and observations. The experimental evidence pages enable the user to view and submit experimental results for individual proteins. The option to post and view comments related to protein-specific information forms a basis for a general discussion forum and motivates scientific exchange and discussion between researchers.

### Posting of laboratory results to the NovelFam3000 system

#### Selection of sample set of genes

To demonstrate the capacity of the NovelFam3000 system to facilitate the inference of protein domain functions, we selected a set of 39 domain families for targeted experimental studies (Table 1). For 25 genes belonging to the target domain families, we confirmed expression in a panel of cell lines, cloned full-length cDNAs, and performed sub-cellular localization analysis [see Additional file 2].

#### Sub-cellular localization

The function of proteins is, in part, defined by the cellular compartment within which they reside. Sub-cellular localization can be determined by visualization of recombinant proteins in amenable cell lines [24,25]. We initiated sub-cellular localization by verifying that a set of predicted human genes were endogenously expressed in human cells. For this purpose, we screened the expression of the 25 selected genes in three human cell lines by reverse transcription polymerase chain reaction (RT-PCR) analysis [see Additional file 3]. The human cell lines, chosen for their suitability for microscopy studies, included the hepatocarcinoma cell line PLC/PRF/5, the glia cell line U333CG/343 MG, and the fibroblast line HF-SV80. Of the 25 candidate genes, 20 were expressed in all three cell lines, three were found to be expressed in two of the three cell lines, and transcripts for two genes were only detected in a single cell line. These observations confirmed the physiological expression of predicted human genes. For sub-cellular screening, full-length human cDNAs were amplified from mRNA and cloned in-frame with an N-terminal FLAG tag. The 25 cloned, FLAG epitope-tagged recombinant proteins were analyzed by immunofluorescence microscopy. Individual transfection of each construct into mammalian cells followed by expression and immunolocalization with monoclonal FLAG-specific antibodies revealed sub-cellular localization of the fusion proteins.

We performed an initial screen to distinguish between cytoplasmic and nuclear localization. This initial classification was followed by counterstaining experiments with multiple sub-cellular markers. Each marker was specific to a sub-cellular compartment, thus facilitating the refined interpretation of previously determined coarse staining patterns. During the primary analysis, we observed six fusion proteins localized to the nucleus, nine proteins in the cytoplasm and six proteins appeared diffusely distributed over the entire cell. Four of the recombinant proteins did not give rise to any detectable staining pattern. All constructs were expressed in the three cell lines to confirm that the observed localization pattern was identical between transfections with the same construct irrespective of the cell type. In the second round of screening, this time limited to PLC/PRF/5 cells, we re-transfected those constructs that had previously given rise to distinct cellular localization patterns, and stained using either antibody markers or specific dyes for cellular structures to confirm co-localization.

All of the expression data and microscopy images from the sub-cellular localization profiling were posted through the laboratory results service of the NovelFam3000 system.

#### Inference of potential domain properties

Within the targeted domain families, we sought to identify intra-family consistencies.

**Figure 2:**
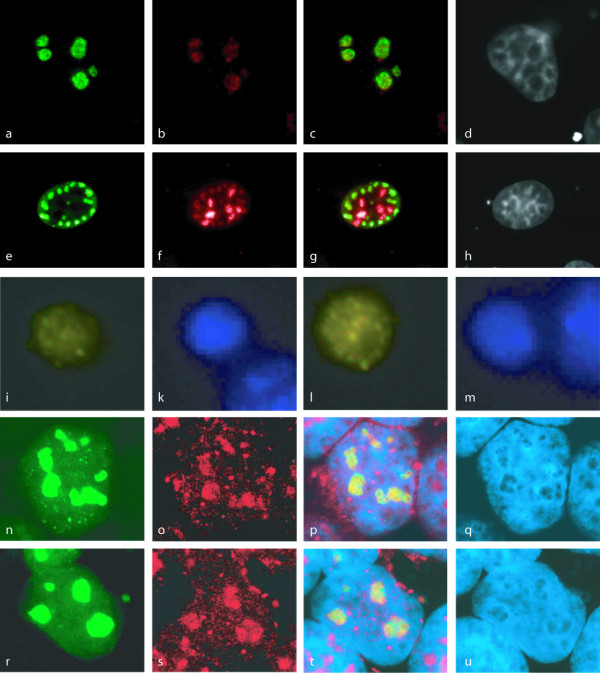
Immunolocalization of protein domain family PF04427 members. Human FLAG-tagged recombinant proteins detected with FLAG-specific antibodies in human PLC/PRF/5 cells (a: BRIX_HUMAN; e: IMP4). Fly His-tagged recombinant proteins detected with His-specific antibodies in fly cells (i: CG11920: l: CG32253). Fly GFP-tagged recombinant proteins detected in human HEK293 cells (n: CG32253; r: CG6712). Fibrillarin staining of nucleoli (b, f, o, s); composite images between proceeding stains (c, g, p, t); DAPI staining of nuclei (d, h, k, m, q, u).

For protein domain family PF004427 (Brix domain), human proteins BRIX_HUMAN and IMP4 localized to the nucleoli (Figure 2). The localization was confirmed by complete co-localization with fibrillarin, a nucleolar-specific marker. To test if additional Brix domain family members localize to the nucleolus and to confirm consistency in sub-cellular localization across organisms, we isolated three *Drosophila *homologs of this domain family (CG32253, CG11920, and CG6712). The cDNAs of the fly genes were cloned into both fly and mammalian expression vectors. All three fly proteins localized to the nucleus in fly cells displaying a consistent nucleolar staining pattern (CG6712 not shown). The expression of the *Drosophila *proteins in human HEK 293 cells was monitored by a C-terminal in-frame GFP tag (CG11920 not shown). The *Drosophila *proteins were found to accumulate in the nucleoli of the human cells, suggesting that the evolutionary conserved protein domain might be implicated in the targeting of these proteins to nucleoli. These results complement published observations for family members in model organisms [26], and in total, suggest that the proteins with the domain perform specific functions in the nucleoli.

**Figure 3:**
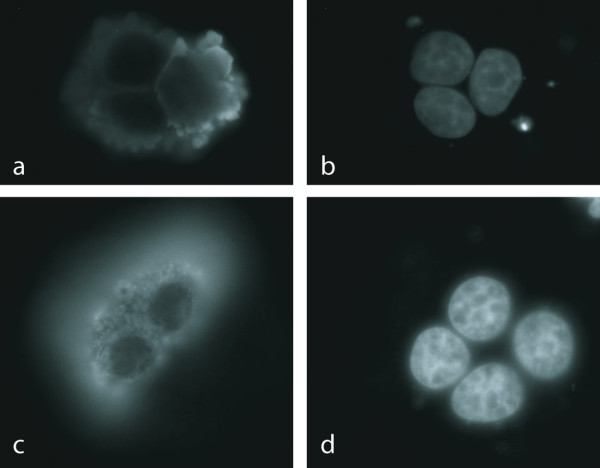
Immunolocalization of protein domain family PF03114 members. FLAG-tagged recombinant proteins detected with FLAG-specific antibodies (a: SH3GL1; c: SH3BP1); DAPI staining of nuclei (b, d). Both proteins show similar vesicular localization patterns. The pattern is distinct from those obtained with multiple cellular markers for vesicle structures.

Intra-family consistency was also observed for protein domain family PF03114 (BAR domain). Member proteins SH3BP1 and SH3GL1 both localized to cytoplasmic vesicles which appeared to merge with the cellular membrane forming protrusions (Figure 3). The familial consistency in the staining patterns observed suggests that the BAR domain is linked to vesicle transport and/or metabolism. The conserved domain might be part of a localization signal that directs the proteins to the observed locations.

**Figure 4:**
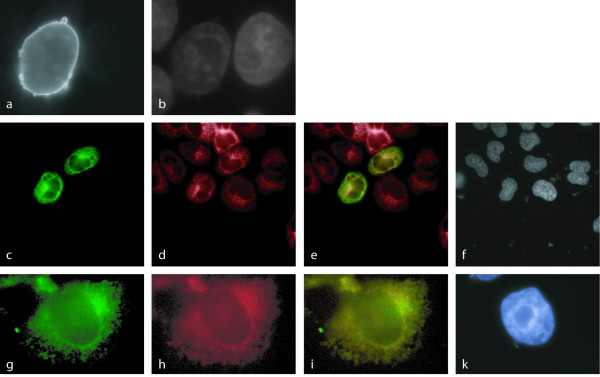
Diverse localization patterns observed by immunolocalization of FLAG-tagged recombinant proteins detected with FLAG-specific antibodies. NP_057480 (HSPC129) of protein domain family PF03031 localizes to the nuclear rim (a), DULLARD is found in the ER (c) as confirmed by calnexin counterstaining (d, e), and CTDSPL is present in mitochondria (g) as confirmed by staining with MitoTracker Red CMX dye (h, i). DAPI staining for nuclei (b, f, k).

We observed an example in which members of the same domain family displayed different, distinct cellular localization patterns (Figure 4). Over-expression of NP_057480 (HSPC129) of domain family PF03031 (NLI interacting factor-like phosphatase domain) in PLC/PRF/5 cells gave a clear and strong staining of the nuclear envelope presenting budding structures. DULLARD of that same domain family displayed a cytoplasmic staining pattern localizing to the endoplasmatic reticulum (ER), as confirmed by calnexin counterstaining. Furthermore, family member CTDSPL co-localized with MitoTracker, a mitochondrion-specific cell-permeant fluorescent dye. These results indicate that the function of this domain is not linked to a specific sub-cellular location.

#### Combining results from multiple sources via NovelFam3000

**Figure 5:**
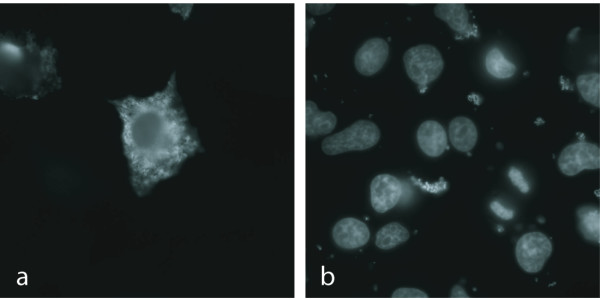
Novel immunolocalization pattern of FLAG-tagged recombinant protein detected with FLAG-specific antibodies (a). DAPI staining for nuclei (b). NP_055268 (CHMP2A, BC-2) of protein family PF03357 forms distinct cytoplasmic structures in PLC/PRF/5 cells.

NP_055268 (CHMP2A, BC-2), a member of protein family PF03357 (SNF7 domain, previously DUF279), gave rise to a unique cytoplasmic staining pattern (Figure 5). We tested hypothetical co-localization with the golgi, the ER, and mitochondria by counterstaining using corresponding markers (data not shown), but could not attribute NP_055268 (CHMP2A, BC-2)'s pattern to any previously defined sub-cellular location. Linking from NovelFam3000 to the Ulysses system, conserved networks in the model organisms suggest that NP_055268 (CHMP2A, BC-2) is a protein involved in pre-vacuolar endosome protein sorting and transport, a hypothesis supported by a previous study [27]. CHMP2A has also been shown to be expressed in the nucleus, possibly having a role in gene silencing [28]. This dual expression pattern is reminiscent of the expression pattern of a related gene, CHMP1, that has been postulated to have a cytoplasmic role in vesicle trafficking, but also a role within the nuclear matrix [29,30].

In addition to the analysis of paralogous human genes (derived by duplication), similarities between family members can be considered across species (orthologs analysis). For those selected proteins present in yeast, we extracted and reviewed sub-cellular localization and interacting protein partners. We show in two examples how the integration of functional data from studies of homologous yeast proteins reveals the broad conservation of function.

Yeast proteins containing the brix domain (PF04427) and their interacting partners have been localized to the nucleolus [31]. Imp4p is a specific component of the U3 snoRNP and is required for pre-18S rRNA processing. Brx1p is implicated in the biogenesis of the 60S ribosomal subunit. The functional differences of human homologs, BRIX_HUMAN and IMP4, are reflected in their observed nucleolar, yet distinct localization patterns (Figure 2).

Protein localization and interaction data from yeast studies complement the observed localization of human NP_057480 (HSPC129) and DULLARD, both from protein family PF03031 (Figure 4). A yeast homolog containing the NIF domain, nem1, is described as a trans-membrane protein localizing to the membranes of the ER and the nucleus [32]. Nem1's specific molecular function is unknown. Protein interaction studies with nem1 have identified three interacting partners (nup84, nup85, nup120), all components of the yeast nuclear pore complex (NPC) [33]. Despite the strong links to the NPC and the localization to the nuclear membrane, we are not convinced that NP_057480 (HSPC129) is a direct component of the vertebrate NPC, since its nuclear rim staining does not show a punctuate pattern – a general feature of NPC elements [34]. Based on the consistency among yeast network members, we identified the human orthologs for the interacting partners. Human NUP107 (related to yeast nup84) supports the NPC link, as this protein is required for the assembly of a subset of "Nup" proteins into the NPC [35]. From the analysis of NP_057480 (HSPC129), its homologs and interacting partners, we hypothesize that this protein is an uncharacterized NPC-associated protein.

## Discussion and conclusion

Based on comparative genome analysis across multiple organisms, protein families have been identified containing domains for which minimal functional annotation is available. From the Pfam database [7] we extracted uncharacterized domain families conserved across vast evolutionary distance, suggesting a well-defined and important cellular role. To elucidate the cellular function of individual proteins, we created the NovelFam3000 system to integrate links to diverse resources, provide an interface for scientific discourse and comments, and house relevant experimental data. As a demonstration, we explored the properties of several domain families and used the NovelFam3000 system to develop data-based inferences.

Existing data mining tools [36-38] collect information and provide ample annotation for predicted genes and gene products from scattered resources. These tools are generally species-specific or concentrate on specific gene properties such as gene expression [39] or gene associations [40]. The NovelFam3000 system is a powerful tool for internet-based information exchange and is unique in its focus on active community participation.

There are aspects of the NovelFam3000 system which are reminiscent of the popular WIKI group communication systems [41]. In comparison, the BioWiki project [42] promises to provide a system for shared content editing, which may be well suited for ontology development projects. While WIKI systems are predicated on user editing of posted information, NovelFam3000 was implemented without the community editing functions, as laboratory data should only be subject to corrections from the source investigator. However, NovelFam3000 does allow for critiques to be posted related to experimental results (subject to editorial review to insure the relevance of postings). In combining a WIKI-like interface with a broad collection of hyperlinks to gene-centric and experimental databases, NovelFam3000 is a unique tool to facilitate inference of protein domain functions.

As the structure of the NovelFam3000 data centre is suitable for any number of projects predicated on the collaborative analysis of sets of genes, the underlying software has been made available on the website – provided as an open-source program with no restrictions on the use or redistribution of the code. Already the software has been revised for use in a large genomics project (the Pleiades Project [43]), with only modest software revision required. Thus, the NovelFam3000 software stands as an important product of this research effort.

We populated the NovelFam3000 experimental data service with an initial panel of results for 25 genes from 39 domain families. The transcripts were detected by RT-PCR and cloned, confirming active transcription. We assigned the proteins to distinct sub-cellular compartments by epitope tagging followed by immunolocalization of the fusion proteins. Consistent localization across members of a protein domain family suggests that the function of the domain is directly linked to location. In some cases, the experimental localization data was complemented by the properties of interacting partners of model organism family members.

The race to functional annotation runs at full speed and the level of cellular characterization of genes is constantly changing. The Gene Characterization Index score displayed in NovelFam3000 provides a dynamic indicator of the status of annotation for each gene. As upward shifts in GCI scores are indicative of advances in the elucidation of the functions of genes in NovelFam3000, dramatic changes will be highlighted on the homepage of the system.

The NovelFam3000 system facilitates community-based curation of gene information.

## Availability

NovelFam3000 is publicly available and can be accessed at . The NovelFam3000 software is available for download without restrictions on the website.

## Authors' contributions

DK participated in the design of the study and generated experimental data. DK, CH, and WWW drafted the manuscript. RMP developed the Gene Characterization Index and contributed to the database design. DA and JL carried out the database development. EH and PR contributed experimental data for model organisms. CH coordinated the generation of the experimental data for the database. ELLS supervised the initial compilation of a collection of novel-domain containing proteins. WWW conceived of the NovalFam3000 database and assisted in the interface design. All authors read and approved of the final manuscript.

## Supplementary Material

Additional File 1 Supplementary Figure. This drawing represents the NovelFam3000 database schema. Each rectangle, labeled with the table name at the top, represents a table in the database. The field names for each table are listed with symbols to the left. Primary Keys are denoted by a yellow key. Foreign Keys are denoted by a red diamond and "(FK)" after the field name. Regular Fields are denoted by a blue diamond. Relations between the tables are indicated by blue lines, with the diamond-end of the line at the referenced table and the other end at the referencing table. The relations are as follows: Rel_01: Comments can be made about a gene; Rel_02: News items can be associated with a gene; Rel_03: Resources can be associated with a gene; Rel_04: Experiments can be associated with a gene; Rel_05: Pfam sequences can be associated with a gene; Rel_06: Pfam sequences can be associated with a Pfam family; Rel_07: An experiment can be associated with multiple instances of experimental data. Click here for file

Additional File 2 Materials and methods. This file contains detailed materials and methods for both the database implementation and the experimental data Click here for file

Additional File 3 Primer sequences. This file contains nucleotide sequences for the gene-specific primers used for RT-PCR amplification of predicted human genes Click here for file

## Figures and Tables

**Table 1:** List of selected domain family members for experimental validation

**Gene name**	**GeneLynx**	**Pfam domain family**	**Domain name**
NP_057480 (HSPC129)	7247	PF03031	NIF
CTDSPL	7767	PF03031	NIF
DULLARD	3595	PF03031	NIF
SH3GL1	5401	PF03114	BAR
SH3BP1	14928	PF03114	BAR
PAPD1(FLJ10486)	13623	PF03828	PAP_assoc
		PB001357	
BRIX_HUMAN	13847	PF04427	Brix
IMP4	3684	PF04427	Brix
NP_115675 (MGC2714)	22343	PF03556	DUF298
		PB003548	
SUPT16H (MGC48972)	10874	PB025336	
		PB006003	
		PB005785	
		PB005762	
		PB005265	
NP_055268 (CHMP2A, BC-2)	8520	PF03357	SNF7
VPS24	11651	PF03357	SNF7
DYM (FLJ20071)	11668	PB011701	
		PB013707	
		PB035957	
KBTBD10 (SARCOSIN)	364	PF07707	BACK
BTBD1	13364	PB006072	
NP_060536 (FLJ10349)	13579	PF03029	ATP_bind_1
NP_057385 (Protein × 0004)	12221	PF03029	ATP_bind_1
NCOA7	24012	PF07534	TLD
		PB007099	
SBNO1	13705	PB012709	
		PB006622	
		PB007895	
		PB008212	
		PB008801	
		PB017731	
		PB017508	
PLCG2	3287	PB010400	
PELI1_HUMAN	17429	PF04710	Pellino
MCTS1	11883	PF01472	PUA
		PB003960	
FAM49B (BM-009)	11348	PF07159	DUF1394
ANKRD13	9528	PB004630	
RABGAP1L (HHL protein, EVI-5 homolog)	14943	PF00566	TBC
		PB008569	
		PB010722	
		PB011629	
		PB008876	
