# Supplementary figures and images for: NovelFam3000 – Uncharacterized human protein domains conserved across model organisms
Source: BMC Genomics. 2006 Mar 13;7:48. doi: 10.1186/1471-2164-7-48 (PMC1440326; doi:10.1186/1471-2164-7-48)

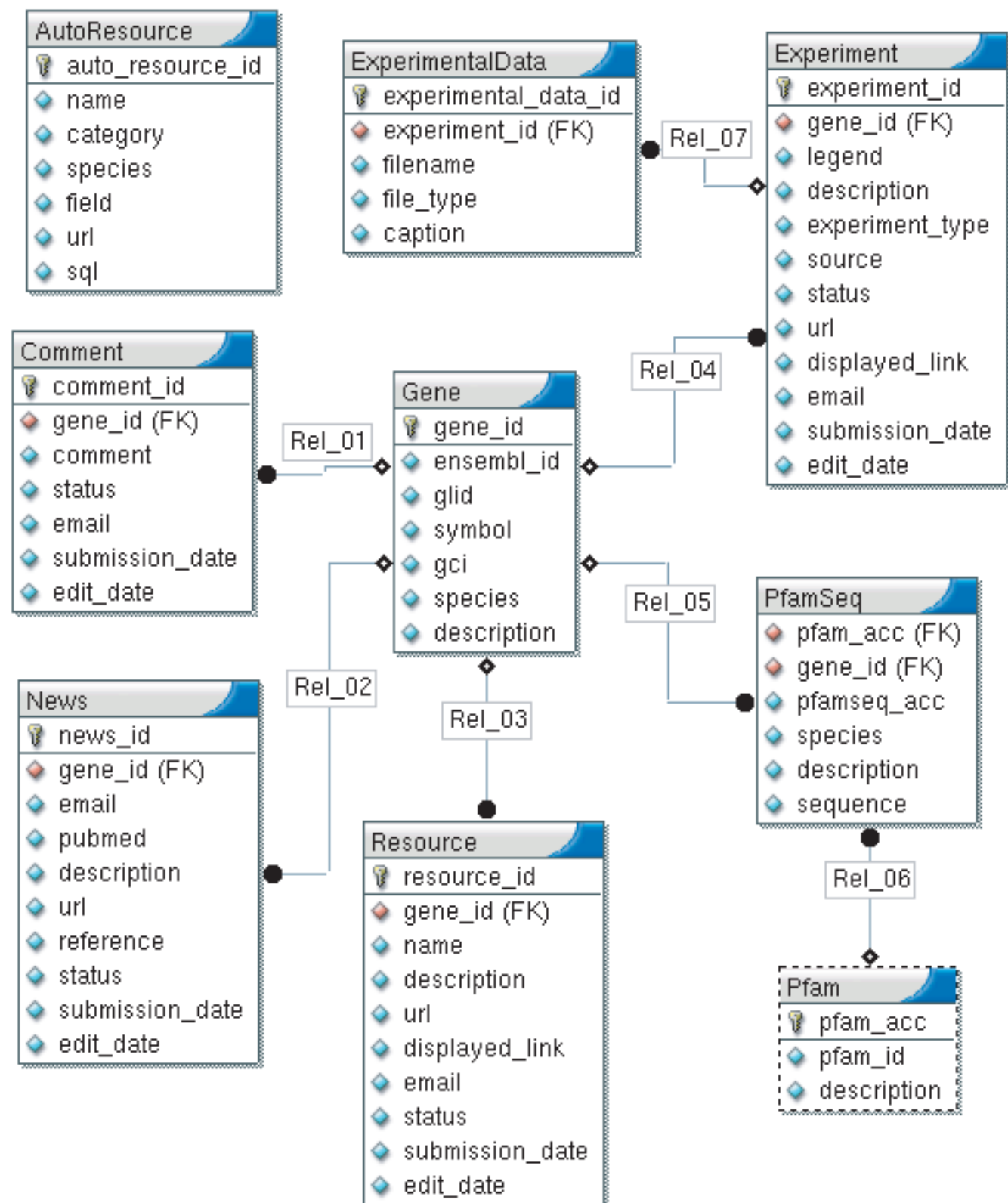

Supplement: Additional File 1 — Supplementary Figure. This drawing represents the NovelFam3000 database schema. Each rectangle, labeled with the table name at the top, represents a table in the database. The field names for each table are listed with symbols to the left. Primary Keys are denoted by a yellow key. Foreign Keys are denoted by a red diamond and "(FK)" after the field name. Regular Fields are denoted by a blue diamond. Relations between the tables are indicated by blue lines, with the diamond-end of the line at the referenced table and the other end at the referencing table. The relations are as follows: Rel_01: Comments can be made about a gene; Rel_02: News items can be associated with a gene; Rel_03: Resources can be associated with a gene; Rel_04: Experiments can be associated with a gene; Rel_05: Pfam sequences can be associated with a gene; Rel_06: Pfam sequences can be associated with a Pfam family; Rel_07: An experiment can be associated with multiple instances of experimental data. [file 1471-2164-7-48-S1.pdf]
