# Supplementary material for: NovelFam3000 – Uncharacterized human protein domains conserved across model organisms
Source: BMC Genomics. 2006 Mar 13;7:48. doi: 10.1186/1471-2164-7-48 (PMC1440326; doi:10.1186/1471-2164-7-48)
Supplement: Additional File 2 — Materials and methods. This file contains detailed materials and methods for both the database implementation and the experimental data [file 1471-2164-7-48-S2.doc]

# Materials and methods

### Protein domain family selection

From the Pfam protein domain database (release 16.0) [7] we extracted all Domains of Unknown Function (DUFs) and Pfam-B domains that were represented in *Homo sapiens*, *Drosophila melanogaster*, and *Caenorhabditis elegans*. Our collection contained 127 DUFs (of which 59 are also present in *Saccharomyces cerevisiae*) and 2785 Pfam-B domains (892 also present in yeast). For further experimental study we selected 39 DUFs (Pfam-A) and Pfam-B domain families – emphasizing uncharacterized domain families represented in the human proteins.

### Database implementation

The NovelFam3000 application is comprised of a MySQL [44] relational database back-end, an Application Programming Interface (API) layer and a web interface. The API was developed in the Perl programming language as a set of object-oriented modules that act as an interface to the database and model the data stored within it. The web interface, also written in Perl, interacts with the API and consists of a main script/module that follows the Common Gateway Interface (CGI) protocol [45]. This main module utilizes the Perl packages CGI::Application (framework for building reusable web-applications) and Template (front-end to the Template Toolkit) which are freely available through the Comprehensive Perl Archive Network (CPAN) [46].

### Cloning of full-length cDNAs

For targeted experimental study the complete ORFs of 25 human genes were cloned using the recombination-based Gateway™ vector system (Invitrogen). The coding regions were amplified by a two-step PCR using gene-specific primers including recombination sites (Interactiva) (see Supplement 2). mRNA was prepared from three human cell lines (PLC/PRF/5, U333CG/343 MG and HF-SV80) using an mRNA Isolation Kit (Boehringer Mannheim). According to the manufacturer’s instructions, the pooled mRNA was reverse transcribed into cDNA (ThermoScript RT-PCR System, Life Technologies) serving as a template for amplification by rTth™ DNA polymerase (PE Biosystems). The obtained PCR products were inserted into the pDONR201 vector (Invitrogen) to generate entry clones as resource vectors for functional studies. All cloned ORFs were subject to full-length sequencing. For sub-cellular localization studies the full-length coding sequences were transferred from the entry clones into the p3xFLAG-CMV-7.1 expression vector (Sigma).

### RT-PCR Expression profiling

cDNA was prepared as described above. PCR amplifications were performed by rTth™ DNA polymerase (PE Biosystems) on single-stranded cDNA in the presence of specific primer pairs (Interactiva). Reactions (50 μl) included rTth™ DNA polymerase with the corresponding XL 3.3 x PCR Buffer II and Mg(OAc)_2_ at a concentration of 1.1mM (PE Biosystems). The cycle settings were as follows: 95°C for 5min, 42 cycles of 94°C for 15s, 54°C for 30s, and 68°C for 3min. At the conclusion a final extension was performed at 68°C for 15min. PCR products were separated on 1.5% agarose gels.

### Transfections

DMEM (supplemented with 10% FBS and 55μg/ml gentamycin) was used to culture PLC/PRF/5 and HF-SV80 cells, whereas U333CG/343 MG cells were cultured in MEM + Earle’s Salts (supplemented with 10% FBS and 55μg/ml gentamycin). Each construct was transiently transfected into all three cell lines using the transfection reagent FuGENE 6™ (Roche) according to the manufacturer’s instructions. Cells were seeded onto cover-slips 17 to 24 hours prior to transfection to achieve 50% confluence. Six to 20 hours after transfection cells were fixed with 2-3.7% paraformaldehyde (PFA), neutralized in 50mM NH_4_Cl, and permeabilized in 0.4% Triton x 100. All transfections were performed at least three times to ensure reproducibility.

### Immunofluorescence staining

Fixed and permeabilized cells were washed in PBS and blocked with 1% BSA in PBS. Primary antibody incubations were performed for 1 hour in 1% BSA in PBS containing anti-FLAG M2 monoclonal antibody (1:400, Sigma) for the N-terminal FLAG fusions. Cells were washed in PBS and incubated for 30 to 60 min with a FITC conjugated sheep anti-mouse secondary antibody (1:75, Sigma). Cells were washed in PBS, subsequently stained with 0.5 μg/ml DAPI, and mounted in vectashield mounting medium (Vector Laboratories).

For counterstaining experiments, primary antibody incubations were performed under the same conditions containing in addition to either anti-FLAG M2 monoclonal antibody mouse (1:400, Sigma) or anti-FLAG polyclonal antibody rabbit (1:180, Sigma) mouse anti-calnexin antibody (1:100, BD Transduction Laboratories), or mouse anti-GM130 (1:250, BD Transduction Laboratories), mouse anti-GRB2 antibody (1:250, BD Transduction Laboratories), human anti-fibrillarin antibody (1:100, Ringertz), mouse anti- EEA1 antibody (1:500, BD Transduction Laboratories), goat anti-vimentin antibody (1:40, Sigma).

Secondary antibody incubations were performed under the conditions described above with either FITC-conjugated sheep anti-mouse secondary anitbody (1:75, Sigma), or Cy2-conjugated donkey anti-rabbit secondary antibody (1:500, Jackson Laboratory) combined with either Cy3-conjugated goat anti-human secondary antibody (1:700, Amersham), or TRITC-conjugated rabbit anti-goat secondary antibody (1:100, Sigma), or Cy3-conjugated donkey anti-mouse secondary antibody (1:1200, Jackson Laboratory). Mitochondria were stained by incubating cells in 100nM MitoTracker Red CMX (Molecular Probes) for 45 min prior to fixation. Lysosomes were stained by incubating cells in 75nM LysoTracker Red DND-99 (Molecular Probes) in DMSO for 45 min prior to fixation. All experiments were at least repeated three times to ensure reproducibility.

### Sub-cellular localization of fly proteins in human cells

Full-length ORFs were reverse transcribed (see above) from polyA+ RNA isolated from adult fly (Clontech). The PCR products were cloned into pDONR201 entry vectors and subsequently transferred into Gateway™ destination vector pcDNA-DEST47 (Invitrogen). DMEM (supplemented with 10% FBS and 10,000U/ml penicillin/streptomycin) was used to culture HEK 293 cells. Each construct was transiently transfected using the transfection reagent lipofectamine 2000™ (Invitrogen) according to the manufacturer’s instructions. Cells were seeded onto cover-slips 17 to 24 hours prior to transfection to achieve 50 to 80% confluence. 30 hours after transfection cells were fixed with 2% PFA and 1.6% sucrose, and were permeabilized in 0.5% Triton x 100.

### Fluorescence microscopy

Protein expression was analyzed at various magnifications using both a Leica DM-RXA and a Leica DM-RA2 fluorescence microscope and digitally imaged with the OpenLab (version 3.0.8.) imaging system (Improvision) using a Hamamatsu C4742-95 digital camera and a Hamamatsu Micro Color Model RGB-MS-C. Final images were prepared using Adobe Photoshop 6.0.

### Sub-cellular localization in fly cells

S2 cells were grown at 25°C in Schneider Drosophila Medium supplemented with 10% FBS. Cells were transfected following the published protocol [47]. The anti-His (C-terminus) antibody (Invitrogen) was used at a 1:1000 dilution. The secondary antibody, anti-mouse Cy3, was used at 1:500 dilution. Stainings were analyzed using a Zeiss fluorescence microscope.
