# Supplementary material for: NovelFam3000 – Uncharacterized human protein domains conserved across model organisms
Source: BMC Genomics. 2006 Mar 13;7:48. doi: 10.1186/1471-2164-7-48 (PMC1440326; doi:10.1186/1471-2164-7-48)
Supplement: Additional File 3 — Primer sequences. This file contains nucleotide sequences for the gene-specific primers used for RT-PCR amplification of predicted human genes [file 1471-2164-7-48-S3.doc]

# Primer sequences

(Recombination sites correspond to first 14 nucleotides for forward and first 12 nucleotides for reverse primers respectively)

Hspc129 protein

5’ AAA AAG CAG GCT CC**A TGA GGC TGA GAA CAC GG** 3’

5’ AGA AAG CTG GGT **AAC AGA GAG AAA ATA AAA GTC C** 3’

Ctdsp-like

5’ AAA AAG CAG GCT CC**A TGG ACG GCC CGG CCA TC** 3’

5’ AGA AAG CTG GGT **AAA AAA ACA AAA CAG GTA GG** 3’

Dullard homolog

5’ AA AAA GCA GGC TCC **ATG ATG CGG ACG CAG TGT C** 3’

5’ A GAA AGC TGG GT**C CTT TCC CCC CCA CCC CAA C** 3’

Sh3-containing grb 2-like 1 protein

5’ AA AAA GCA GGC TCC **ATG TCG GTG GCG GGG CTG** 3’

5’ A GAA AGC TGG GT**C CCA GAT AAG CCC CCC CAC CC** 3’

Sh3 domain-binding protein 1

5’ AAA AAG CAG GCT CC**A TGG ACA AGC GGG TGA AG** 3’

5’ AGA AAG CTG GGT **AGT TGG TCT CTG AGG CAA** **G** 3’

Hypothetical protein flj10486

5’ AAA AAG CAG GCT CC**A TGG CGG TTC CCG GCG TG 3’**

5’ AGA AAG CTG GGT **TCA GTT TTT CCA AGT AAG TCC ACA G** 3’

Ribosome biogenesis protein brix

5’ AAA AAG CAG GCT CC**A TGG CGG CAA CCA AGA GGA AAC** 3’

5’ AGA AAG CTG GGT **ATT TTG TTT TCC CAC TGT CCA TCC** 3’

U3 snorp protein 4 homolog

5’ AA AAA GCA GGC TCC **ATG CTG CGC CGC GAG GCC** 3’

5’ A GAA AGC TGG GT**A GTT CCA CAT CCC AGT TCA GCA GG** 3’

Mgc2714

5’ AAA AAG CAG GCT CC**A TGC CGG TGA AG** 3’

5’ AGA AAG CTG GGT **GGA CTT TCG CCA CTC AAC** 3’

Mgc48972

5’ AAA AAG CAG GCT CC**A TGG GCC AGT GTG TCA CC** 3’

5’ AGA AAG CTG GGT **ATC CTT GCT GCT GCT CCT G** 3’

Bc-2 protein

5’ AA AAA GCA GGC TCC **ATG GAC CTA TTG TTC GGG** 3’

5’ A GAA AGC TGG GT**G GCA CTC AGT CCC TCC GCA G** 3’

Cgi-149 protein

5’ AAA AAG CAG GCT CC**A TGG GGC TGT TTG GAA AG** 3’

5’ AGA AAG CTG GGT **AAA AGA GAC AAA CAG AAC** 3’

Hypothetical protein flj20071

5’ AAA AAG CAG GCT CC**A TGG GAT CGA ATA GCA GC** 3’

5’ AGA AAG CTG GGT **AAT GAC ATT TTA GGT CTT GTG** 3’

Sarcosin

5’ AA AAA GCA GGC TCC **ATG CAA CTG TCT CCA CAG GA** 3’

5’ A GAA AGC TGG GT**T TCA CCA AAC AAA CCA CCT CC** 3’

Btb and kelch domain-containing protein 1

5’ AA AAA GCA GGC TCC **ATG GCC TCA CTC GGG CCT GCC** 3’

5’ A GAA AGC TGG GT**G CCT ATT TGC CCA TCT CAC CTA AC** 3’

Hypothetical protein flj10349

5’ AA AAA GCA GGC TCC **ATG GCA GGG GCC GCT CCG A** 3’

5’ A GAA AGC TGG GT**C AGA GCT GAA TAT CCC CTT GCC** 3’

Protein x 0004

5’ AA AAA GCA GGC TCC **ATG CCT CGG TAT GCG CAC TGC** 3’

5’ A GAA AGC TGG GT**T TCA GGC ATG AGG CTG ATA AAG AAC** 3’

Hypothetical protein

5’ AAA AAG CAG GCT CC**A TGG GTG CTG CAC ACA GTG TTG G** 3’

5’ AGA AAG CTG GGT **CAT AAA CCA AAT CGT CCC CCT CC** 3’

Sbno1

5’ AA AAA GCA GGC TCC **ATG GTG GAG CCA GGG CAA GAT TTA** 3’

5’ A GAA AGC TGG GT**G TGG TCT TTT CTA ATT AAC CAG GG** 3’

Phospholipase c-gamma 2

5’ AA AAA GCA GGC TCC **ATG TCC ACC ACG GTC AAT GTA GAT T** 3’

5’ A GAA AGC TGG GT**C CTT ACA CAC ATA CCC CAG CTT C** 3’

Pellino protein homolog 1

5’ AAA AAG CAG GCT CC**A TGT TTT CTC CTG ATC AAG** 3’

5’ AGA AAG CTG GGT **CAA AAC AAA CAC CTT TCA** **C** 3’

Malignant t cell amplified sequence 1

5’ AA AAA GCA GGC TCC **ATG TTC AAG AAA TTT GAT GAA AAA G** 3’

5’ A GAA AGC TGG GT**C AGC ATA ACC ACA GGC ATT ATC TTC** 3’

Hypothetical protein bm-009

5’ AA AAA GCA GGC TCC **ATG CAG** **AAG GCA TCT TGG AG** 3’

5’ A GAA AGC TGG GT**T CCA CAC TAT TAG GAG GGT TG** 3’

Ny-ren 25

5’ AAA AAG CAG GCT CC**A TGT CCT CGG CCT GCG AC** 3’

5’ AGA AAG CTG GGT **CAC TTG TCT TGT TAT CAC TCC** 3’

Hhl protein (evi-5 homolog)

5’ AAA AAG CAG GCT CC**A TGT TAT TAA GCC CAG GTC G** 3’

5’ AGA AAG CTG GGT **CAC TTT CTT TTT CCC CTC TCT TTC** 3’
